# 350. Unnecessary viral blood tests reduction and costs saving by using an automated test request blocking system

**DOI:** 10.1093/ofid/ofad500.421

**Published:** 2023-11-27

**Authors:** Daniella Rahamim-Cohen, Lea Valinsky, Anat Beit Or, Orna Shamai-Lubovitz, Moshe Fisher, Josef Azuri, Shirley Shapiro Ben David

**Affiliations:** Maccabi Healthcare Services, Tel Aviv, Tel Aviv, Israel; Maccabi Healthcare Services, Tel Aviv, Tel Aviv, Israel; Maccabi Healthcare Services, Tel Aviv, Tel Aviv, Israel; Maccabi Healthcare Services, Tel Aviv, Tel Aviv, Israel; Maccabi Healthcare Services, Tel Aviv, Tel Aviv, Israel; Maccabi Healthcare Services, Tel Aviv, Tel Aviv, Israel; Maccabi Healthcare Services, Tel Aviv, Tel Aviv, Israel

## Abstract

**Background:**

As demands and costs rise across all aspects of healthcare, blood tests form a significant part of existing health expenses. Laboratory analysis provides a vital tool for the evaluation and monitoring of patients with suspected infectious diseases in the community, but are occasionally ordered without accurate clinical indications, resulting in unnecessary expense, workload and patient distress. Here we examined the economic utility of integrating a rule engine system, used in this context to block unnecessary infectious disease laboratory tests.

**Methods:**

An automated blocking system for blood tests, including tests for viral diseases, was designed and implemented within the electronic medical record (EMR) by Maccabi Healthcare Services, a health maintenance organization in Israel, serving over 2.5 million members, nationwide. The system blocks requests for tests identified as unnecessary, usually based on a previous result. Specifically, the system blocks blood tests for the following viral diseases: Hepatitis C viral load and serology for: EBV, CMV, Rubella, Varicella Zoster and Hepatitis B. Upon provider request, a non- authorized test can be re-evaluated by an infectious diseases specialist (Figure 1). Test costs were assessed using the standard Israel Ministry of Health price list in Israeli currency (New Israeli Shekel-NIS) and are expressed in 2023 USD (1$=3.62 NIS, according to the currency exchange rate on May 2, 2023).

**Figure 1:**
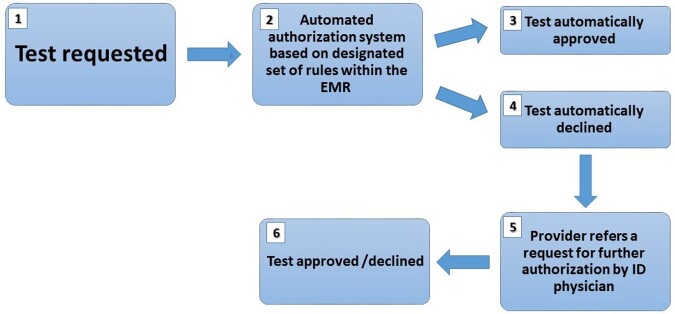
Automated test request blocking system workflow. EMR Electronic Medical Record, ID Infectious Disease

**Results:**

During 2022, 407,403 requested viral blood tests were recoded (Table 1). Of them, 302,322 (74.2%) were automatically approved. Of those blocked, only a small proportion were requested for further authorization (between 0.15-1.1%). The calculated savings for declined tests is approximately 5.8 million USD.

**Figure:**
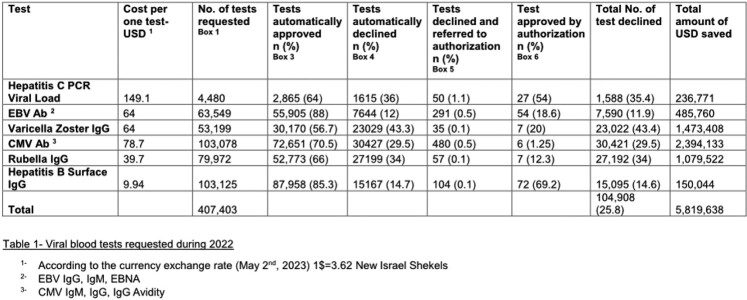

**Conclusion:**

This automated authorization system for certain viral blood tests resulted in an improved clinical decision process by blocking unnecessary tests and led to substantial monetary saving without sacrificing physician autonomy.

**Disclosures:**

**All Authors**: No reported disclosures

